# Visual perception affected by motivation and alertness controlled by a noninvasive brain-computer interface

**DOI:** 10.1371/journal.pone.0188700

**Published:** 2017-12-21

**Authors:** Vladimir A. Maksimenko, Anastasia E. Runnova, Maksim O. Zhuravlev, Vladimir V. Makarov, Vladimir Nedayvozov, Vadim V. Grubov, Svetlana V. Pchelintceva, Alexander E. Hramov, Alexander N. Pisarchik

**Affiliations:** 1 Yuri Gagarin Technical State University of Saratov, Politehnicheskaya, 77, 410054 Saratov, Russia; 2 Center for Biomedical Technology, Technical University of Madrid, Campus Montegancedo, 28223 Pozuelo de Alarcon, Madrid, Spain; University of Illinois at Urbana-Champaign, UNITED STATES

## Abstract

The influence of motivation and alertness on brain activity associated with visual perception was studied experimentally using the Necker cube, which ambiguity was controlled by the contrast of its ribs. The wavelet analysis of recorded multichannel electroencephalograms (EEG) allowed us to distinguish two different scenarios while the brain processed the ambiguous stimulus. The first scenario is characterized by a particular destruction of alpha rhythm (8–12 Hz) with a simultaneous increase in beta-wave activity (20–30 Hz), whereas in the second scenario, the beta rhythm is not well pronounced while the alpha-wave energy remains unchanged. The experiments were carried out with a group of financially motivated subjects and another group of unpaid volunteers. It was found that the first scenario occurred mainly in the motivated group. This can be explained by the increased alertness of the motivated subjects. The prevalence of the first scenario was also observed in a group of subjects to whom images with higher ambiguity were presented. We believe that the revealed scenarios can occur not only during the perception of bistable images, but also in other perceptual tasks requiring decision making. The obtained results may have important applications for monitoring and controlling human alertness in situations which need substantial attention. On the base of the obtained results we built a brain-computer interface to estimate and control the degree of alertness in real time.

## Introduction

The brain is often considered as a complex network of interacting units (neurons) [1–3], which cooperative dynamics causes different types of cognitive activity, e.g., the formation of memory traces [4, 5], information processing [6, 7], spatial orientation [8, 9], intelligence [10, 11], etc. These types of brain activity were extensively studied by the scientific community [4–11] due to their great importance. The information processing in the brain is composed of the following steps: acquisition of external data (stimuli), their analysis, and the brain response. Each of these steps is characterized by a simultaneous activation of certain brain areas which interact with each other due to their functional connection.

Cognitive brain function is usually affected by individual human physiological features, i.e., the same type of human activity can be associated with different scenarios of cognitive brain processes, depending on the motivation, alertness, health status, weariness, etc. of the person [12–15]. Therefore, along with the knowledge of basic features of the brain activity in solving particular tasks, it is of great practical importance to study the influence of the human factors, such as responsibility, motivation, attention, and stress [16, 17]. In this context, the neurophysiological research of visual perception focused on the detection of brain activity and interactions between different brain regions [18–22] demonstrated an increased activity in various brain areas, especially in the occipital cortex [18, 19, 23]. For example, the functional magnetic resonance data [20] indicated a symmetrical activation of the premotor and parietal areas during perception. Wang et al. [22] observed that perception of bistable images involved many higher-order frontoparietal and temporal regions. The connectivity between the anterior and posterior regions of the superior parietal lobule and their interplay with regions of sensory motor and associative cortex were also detected in the process of perception of ambiguous images [21].

At the same time, visual perception was shown to be highly affected by the human factor, such as motivation, alertness, attention, responsibility, health conditions, etc. [24–26]. The influence of attention on perception was studied using event related potential (ERP) recordings [27] in electroencephalographic (EEG) or magnetoencephalographic (MEG) data by averaging over a large number of EEG (or MEG) traces associated with the perception of stimuli. The ERP approach was widely used for the analysis of visual attention, in particular, for studying selective attention of humans [28], including neural mechanisms of spatial selective attention [29], effects of mental fatigue on the attention [30], and effects of attention on visual lexical categorization [31]. According to Elmer [32], a specific brain response to a particular stimulus is too small to be distinguished in a single EEG. Even though the findings based on the ERP are useful for identification of characteristic features of the brain activity during a long experimental session, they are useless for the analysis of its variation in time. To study how motivation and attention affect alpha activity, Vázquez et al. [33] applied temporal spectral evolution technique. They discovered that increasing attention resulted in a decrease in alpha-wave power. Later, it was found that alpha-band activity was related to anticipatory and temporal attention [34, 35]. Recently, the suppression of alpha activity was connected to sensory attention [36, 37]. It was also shown that changes in attention induced by special auditory stimuli can modulate alpha energy [38–40].

Many scientists emphasized that for understanding cognitive mechanisms responsible for alertness we should explore the brain activity in a wide frequency range, simultaneously analyzing different rhythms (delta, alpha, and beta) [41–43]. According to Refs. [42, 43], such an approach would allow simultaneous observation of different states of the neuronal network, which in turn would be very useful for understanding not only perception, but also other types of cognitive brain activity. Despite the large number of publications concerning alpha activity, there are very few papers devoted to the relation between alpha waves and motivation. In particular, some researchers associated different types of motivation with alpha asymmetries [44, 45].

In this work we study the influence of motivation and alertness on the processing of visual perceptual tasks requiring decision making. These tasks are suitable for the estimation of the degree of alertness, because they need quick attention on the image presented, followed by cognitive brain processing. We propose possible scenarios of the brain cognitive activity associated with visual perception of ambiguous images for groups of participants with different degrees of motivation and alertness. In our experiments we use ambiguous (bistable) stimuli because they are a very useful tool for studying the decision-making process [46–49]. We picked the Necker cube as the research object [50]. The significance of the Necker cube task in studying the decision-making process was emphasized by many researchers, who discussed underlying neural mechanisms responsible for multistable perception. According to Zeki [51], the perceptual system organizes sensory information in the coherent interpretation of the outside world. Therefore, one can expect that if there is more than one plausible interpretation of the sensory evidence, the basic mechanisms of the perceptual decision-making, underlying visual perception, can be responsible for other decision-making tasks [48, 52, 53].

First, the perception of bistable objects requires strong visual attention [54], second, the complexity of the visual task can be easily controlled by varying the degree of ambiguity, and finally, the bistable perception includes the cognitive activity associated with a decision-making process [22] similar for different persons. At the same time, we expect that the results obtained in the experiments with bistable stimuli can be generalized for other perception tasks.

We hypothesize that the brain can process visual stimuli in different ways (scenarios) depending on the degree of alertness of the observer, which, in turn, can be affected by the motivation of the subject and complexity of the task. In many previous papers, the association of alpha and beta waves with attention was mentioned. In particular, it was found that the intensity of alpha rhythm is closely linked to the suppression and selection of attention [55, 56]. Moreover, a relation between changes in alpha oscillations in visual cortex and attention performance was found [57]. A correlation of beta activity with visual attention was also found [58, 59]. Based on the previous results, we consider the features of EEG signals in alpha band (8–12 Hz) and high-frequency beta band (20–30 Hz) before and during the presentation of each stimuli in order to find appropriate criteria for the classification of each perception into one or another scenario. If the perception of individual stimuli could be effectively classified in different scenarios according to the attention, we would be able to identify the ratio between the occurrence of one or another scenario in real time based on the spectral properties of multichannel EEGs. [60]. The relationship between different scenarios would allow us to estimate the degree of alertness of the observer during visual perception and analyze the effect of motivation and task complexity, which would be impossible to do using approaches described in Refs. [55–59] or methods based on the event-related potentials (ERP) [28–31].

In order to test our hypothesis we carry out series of electrophysiological experiments. First, bistable images are presented to randomly selected subjects in similar conditions. Then, we make statistical analysis of the brain activity of every subject in alpha and beta bands during the transition from a “ready” state (before image exposition) to the perception (during image exposition). According to the relationship between alpha and beta activity, we divide the participants into two groups according to two distinct scenarios. Then, based on the obtained results we formulate the features of the first and the second scenario and calculate the ratio between individual perceptions, which satisfies these scenarios for every participant. After that, we perform similar experiments for two groups of subjects, financially motivated participants and unpaid volunteers. Using the recorded EEG data we calculate the number of events belonging to the first and the second scenarios and compare these values for two groups. Next, we carry out experiments with each group of participants by presenting images with high and low degrees of ambiguity, and analyze the relation between the first and the second scenarios. Finally, we develop a brain-computer interface and demonstrate how the degree of alertness can be estimated in real time and how it can be affected by the feedback using external stimulation.

## Materials and methods

### Participants

Forty healthy subjects from a group of students, researchers and staff of the Yuri Gagarin State Technical University of Saratov, males and females, between the ages of 20 and 43 with normal or corrected-to-normal visual acuity participated in the experiments. All of them provided informed written consent before participating in the experiment. The experimental studies were performed in accordance with the Declaration of Helsinki and approved by the local research ethics committee of the Yuri Gagarin State Technical University of Saratov.

### Stimuli

**Fig 1:**
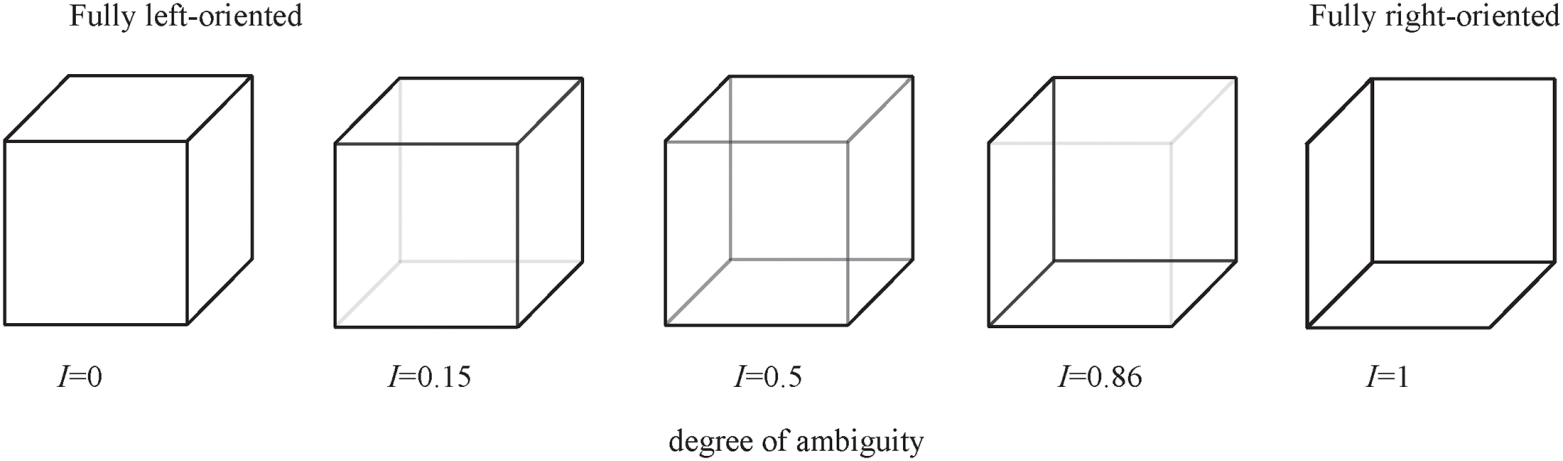
Examples of Necker cube images. The ambiguity of the Necker cube is controlled by contrast parameter *I*. The left-hand image with *I* = 0 corresponds to the fully left-oriented cube, whereas the right-hand image with *I* = 1 to the fully right-oriented cube. The middle image with *I* = 0.5 has the highest ambiguity.

In our experiments we used the Necker cube [50] (Fig 1), the popular object of many psychological experiments [23, 61–64] and theoretical models [62, 65, 66]. This ambiguous image is a cube with transparent faces and visible ribs; an observer without any perception abnormalities sees the Necker cube as a 3D-object due to the specific position of the cube’s ribs. Bistability in perception consists in the interpretation of this 3D-object as to be either left- or right-oriented depending on the constrast of different inner ribs of the cube. The contrast *I* ∈ [0, 1] of the three middle lines centered in the left middle corner was used as a control parameter. The values *I* = 1 and *I* = 0 correspond, respectively, to 0 (black) and 255 (white) pixels’ luminance of the middle lines. Therefore, we can define a contrast parameter as *I* = *y*/255, where *y* is the brightness level of the middle lines using the 8-bit grayscale palette.

### Experimental procedure

While observing the most ambiguous image (central cube in Fig 1) for a prolonged time, the mean duration of a particular interpretation of the cube orientation (left or right) is known to vary from one second to several minutes depending on the observer and stimulus conditions (see, e.g. [67]), whereas the mean response times are rather consistent and varied only by a few hundred milliseconds (see, e.g. [68]). In order to fix the first impression of the person and avoid switches between two possible percepts, we presented the Necker cube images with different wireframe contrasts (as those shown in Fig 1) for short time intervals, each lasting between 1.0 and 1.5 seconds. Such short durations of the stimuli were chosen to reduce the stabilization effect [69, 70], because the probability of the image interpretation, persisting until the subsequent presentation, strongly depends on the duration of the previously observed image. There was a high probability for a perceptual configuration to persist to the next stimulus presentation only when it was seen consistently for a relatively long time until the stimulus disappeared. For the Necker cube the required time of the consistent observation is known to be about 1 second [69, 70]. Although the “memory” effect cannot be completely avoided, it can be significantly diminished by making the length of the stimulus exhibition *ν* shorter than 1.5 seconds. Moreover, a random variation of the control parameter *I* also prevents the perception stabilization. Lastly, to draw away the observer’s attention and make the perception of the next Necker cube independent of the previous one, different abstract pictures were exhibited for about *η* = 5.0 − 5.5 seconds between subsequent demonstrations of the Necker cube images.

All participants were instructed to press either the left or right key depending on their first impression of the cube orientation at each presentation. The whole experiment lasted around 45–50 min for each participant, including short recordings of the brain background activity before and after the stimuli presentation. During experimental sessions, the cubes with different *I* were randomly presented (each configuration for about 100 times) and the electrical brain activity was recorded using the electroencephalographic recorder Encephalan-EEGR-19/26 (Medicom MTD, Russia) which provided simultaneous registration of up to 20 EEG channels and a two-button input device. The monopolar registration method and the classical ten-twenty electrode system were used. The gray-scale images were demonstrated on the 24” BenQ LCD monitor with a resolution of 1920 × 1080 pixels and a refresh rate of 60 Hz. The subject was located at a distance of 70–80 cm from the monitor with a visual angle of approximately 0.25 rad.

The experimental procedure was organized as follows. In the first stage, 10 subjects were randomly selected among 30 participants. In this stage, the motivation factor was not considered and therefore there was no payment. During the experiment, the Necker cubes with different rib contrasts were presented about 400 times to each subject. In this experiment we used six unique stimuli, for which the value of the contrast parameter of internal ribs was randomly chosen from the set *I* = (0.15, 0.35, 0.45, 0.55, 0.65, 0.85). As the result, each cube with a particular contrast was presented more than 60 times.

In the second stage, the remaining 20 subjects were divided into two equal groups, 10 financially motivated and 10 non-motivated. The members of the motivated group had a concrete task: try to identify all the cubes as correctly as possible. The members of the second group were unpaid volunteering students and staff, who participated in experimental sessions daily at random hours. Similar to the first stage, all participants were subjected to 40-min sessions during which 400 Necker cube images were presented.

In the third stage, two sessions (20 minutes each) were organized for 10 extra unpaid volunteers, during which 500 Necker cube images were presented. The design of these sessions was practically the same, but the contrast parameter *I* was different. In one session only cubes with low ambiguity (*I* = 0.15 and *I* = 0.85) were presented, whereas in the other session only cubes with high ambiguity *I* = (0.4, 0.5, 0.6) were shown. In all sessions the cubes were randomly mixed, and each contrast was used about 120 times in the former session and 80 times in the latter session. In this stage, there was no payment because we expected that the motivation factor in this experiment would have a stronger effect on the EEG than the task complexity. Since both factors, motivation and complexity, lead to increasing alertness, the paid subjects demonstrated higher alertness in both sessions as compared with unpaid participants. This is not convenient for this experiment because task complexity is supposed to be of lower significance.

Finally, in the fourth stage, we tested the efficiency of the created brain-computer interface (BCI). Three unpaid volunteers participated in this experiment. For each subject, the whole experiment lasted 12 minutes and was divided into three sessions (4 minutes each). During the first session, approximately 30 cubes were presented to each subject seated in comfortable conditions in the absence of any additional tasks. Before the experiment, every participant was instructed to focus his/her attention on the appearing cubes and press the appropriate joystick button according to the apparent cube orientation. The second session included the external influence on the subject in the form of an additional cognitive task. Namely, during the cube presentation the subject was required to perform a recursive arithmetic operation. Specifically, he/she was asked to subsequently subtract different numbers from a given large number. During the third session, a feedback control was performed in the form of a short sonic tone each time the degree of the subject’s alertness (estimated by the BCI) fell below the threshold value, so that the subject needed to concentrate on the interpretation of the presented visual stimuli (Necker cubes).

### EEG analysis

We analyzed the EEG signals recorded by five electrodes (O_1_, O_2_, P_3_, P_4_, P_*z*_) placed on the standard positions of the ten-twenty international system [71], using the continuous wavelet transformation. The wavelet energy spectrum $E^{n}\left(f,t\right)=\sqrt{W_{n}{\left(f,t\right)}^{2}}$ was calculated for each EEG channel *X*_*n*_(*t*) in the frequency range *f* ∈ [1, 30] Hz. Here, *W*_*n*_(*f*, *t*) is the complex-valued wavelet coefficients calculated as [72]

$$\begin{array}{c} W_{n}\left(f,t\right)=\sqrt{f}\int_{t-4/f}^{t+4/f}X_{n}\left(t\right)\psi^{*}\left(f,t\right)dt, \end{array}$$ (1)

where *n* = 1, …, *N* is the EEG channel number (*N* = 5 being the total number of channels used for the analysis) and * defines the complex conjugation. The mother wavelet function *ψ*(*f*, *t*) is the Morlet wavelet often used for the analysis of neurophysiological data defined as [72]

$$\begin{array}{c} \psi \left(f,t\right)=\sqrt{f}\pi^{1/4}e^{j\omega_{0}f\left(t-t_{0}\right)}e^{f{\left(t-t_{0}\right)}^{2}/2}, \end{array}$$ (2)

where *ω*_0_ = 2*π* is the wavelet parameter.

**Fig 2:**
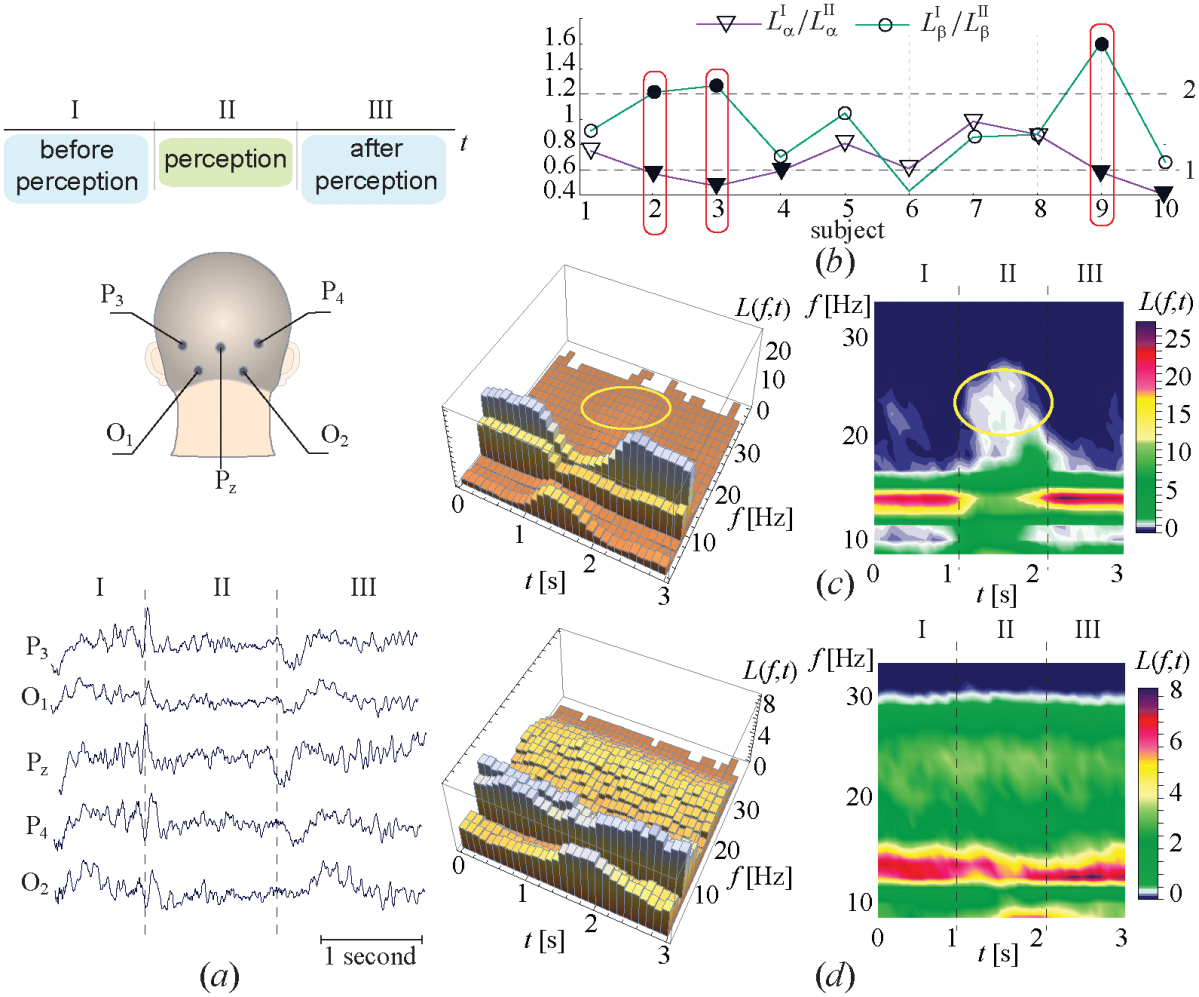
Experimental observation. (a) The scheme of the electrode position the typical set of registered EEG traces. Different segments of the EEG recording are named I, II, III, which correspond, respectively, to the 1-sec time interval preceding the cube presentation (*before perception*), ∼ 1-sec interval of the cube observation (*perception*), and 1-sec interval after the cube observation (*after perception*) and (b) The values of $L_{\alpha }^{I}/L_{\alpha }^{II}$ (triangles) and $L_{\beta }^{I}/L_{\beta }^{II}$ (circles) illustrating the relation between the power of alpha and beta waves in intervals I and II obtained by the statistical analysis of the 40-min experimental session of each of the 10 subjects. The horizontal dashed lines indicate threshold values defining a > 40% decrease of alpha-activity (line 1) and a > 20% increase of beta-activity (line 2) used to identify different perception scenarios. The solid red boxes highlight the subjects (2,3,9) following the first scenario. Other subjects are associated with the second scenario. (c,d) 3-D histograms illustrating the distribution of the statistical measure *L*(*f*, *t*) calculated by Eq (3) which indicates the location of the maximal spectral component during the 40-min session for two subjects demonstrating the (c) first (subject #9) and (d) second (subject #7) perception scenarios.

In the first experiment, we estimated the value of frequency *f*_*max*_(*t*) corresponding to the maximum energy in the wavelet spectrum using Eq (1), for every moment of time. The whole experimental series were split into number *N*_*tr*_ 3-sec trials associated with perception of each individual stimulus. Each trial consisted of three subsequent segments: (I) before image presentation, (II) during presentation, and (III) after presentation, as illustrated in Fig 2A. Then, every trial was split into $N_{\delta_{t}}=15$ time intervals of *δ*_*t*_ = 0.2 sec long, and its power spectrum was split into $N_{\delta_{f}}=15$ bands of *δ*_*f*_ = 0.2 Hz width. For the considered time-frequency plane (*t* ∈ [0, 3] s, *f* ∈ [1, 30] Hz) the distribution of frequency *f*_*max*_ corresponding to the maximum energy was calculated as follows

$$\begin{array}{c} L\left(f,t\right)=\sum_{N_{tr}}\sum_{N_{\Delta_{t}}}\sum_{N_{\Delta_{f}}}\gamma ,\gamma =\{\begin{array}{cc} 1, & f_{max}\left(t\right)\in \delta f\;\wedge \;t\in \delta t\\ 0, & \text{otherwise}. \end{array} \end{array}$$ (3)

In order to quantitatively characterize the distribution *L*(*f*, *t*), for each participant the ratios $L_{\alpha }^{I}/L_{\alpha }^{II}$ and $L_{\beta }^{I}/L_{\beta }^{II}$ were calculated as

$$\begin{array}{c} L_{\alpha ,\beta }^{I,II}=\int_{\Delta t_{I,II}}\int_{\Delta f_{\alpha ,\beta }}L\left(f^{\prime },t^{\prime }\right)df^{\prime }dt^{\prime }, \end{array}$$ (4)

where Δ*f*_*α*,*β*_ is the range of alpha and beta activities and Δ*t*_*I*,*II*_ is the duration of segments I and II.

Depending on the values $L_{\alpha }^{I}/L_{\alpha }^{II}$ and $L_{\beta }^{I}/L_{\beta }^{II}$ two different scenarios were identified. The first scenario (Sc.1) was characterized by a significant decrease in the alpha energy during the segment II (perception) with a simultaneous relatively high increase in the beta energy. The second scenario (Sc.2) was distinguished by a strong contribution of alpha-rhythm and much lower pronounced generation of beta-rhythm during all segments. A more detailed analysis of the spectral properties associated with the first and second scenarios was performed for three frequency bands: Δ*f*_*δ*_ = [1 − 4] Hz (*δ*-rhythm), Δ*f*_*α*_ = [8 − 12] Hz (*α*-rhythm), and Δ*f*_*β*_ = [20 − 30] Hz (*β*-rhythm), corresponding to typical patterns of the human cognitive activity. The EEG power spectrum was characterized by the location of the dominant (most pronounced) spectral components. In particular, the first (maximal) spectral component in the *n*-th EEG channel occurred at frequency $f_{1}^{n}\left(t\right)$ at which the global maximum $E^{n}\left(f_{1}^{n}\left(t\right),t\right)$ took place. Respectively, the second, third, …, *M*-th spectral components appeared at frequencies $f_{2,\ldots ,M}^{n}\left(t\right)$, where $E^{n}\left(f_{2,\ldots ,M}^{n}\left(t\right),t\right)$ exhibited subsequent local maxima.

Using the values $f_{2,\ldots ,M}^{n}\left(t\right)$ the EEG spectral properties were characterized by spectral coefficients $F_{\alpha ,\beta ,\delta }^{n}\left(t\right)$ calculated for each channel at every moment of time

$$\begin{array}{c} F_{\alpha ,\beta ,\delta }^{n}\left(t\right)=\sum_{j=1}^{M}\Theta_{\alpha ,\beta ,\delta }^{n}\left(j,t\right),\;\Theta_{\alpha ,\beta ,\delta }^{n}\left(j,t\right)=\{\begin{array}{ccc} 1/j, & \text{if} & f_{j}^{n}\in \Delta f_{\alpha ,\beta ,\delta },\\ 0, & \text{if} & f_{j}^{n}\notin \Delta f_{\alpha ,\beta ,\delta }. \end{array} \end{array}$$ (5)

The obtained spectral coefficients $F_{\alpha ,\beta ,\delta }^{n}\left(t\right)$ were averaged over all channels and time intervals for each segment (I, II, III) as follows

$$\begin{array}{c} {\langle F_{\alpha ,\beta ,\delta }\rangle }_{\Delta t_{\text{I}}{}_{,\text{II},\text{III}}}=\frac{1}{N}\sum_{n=1}^{N}\underset{\Delta t_{\text{I}}{}_{,\text{II},\text{III}}}{\int }F_{\alpha ,\beta ,\delta }^{n}\left(t^{\prime }\right)dt^{\prime }. \end{array}$$ (6)

Then, for every subject the values of ${\langle F_{\alpha ,\beta ,\delta }\rangle }_{\Delta t_{\text{I},\text{II},\text{III}}}$ were averaged over *K* = 400 trials associated with individual perceptions:

$${\langle {\bar{F}}_{\alpha ,\beta ,\delta }\rangle }_{\Delta t_{\text{I}}{}_{,\text{II},\text{III}}}=\frac{1}{K}\overset{K}{\underset{i=1}{\sum }}{\langle F_{\alpha ,\beta ,\delta }\rangle }_{\Delta t_{\text{I},\text{II},\text{III}}^{i}},$$ (7)

where $\Delta t_{I}^{i}$, $\Delta t_{\text{II}}^{i}$, $\Delta t_{\text{III}}^{i}$ are the time intervals of segments I, II, III, associated with the *i*-th perception event, and $\bar{F}$ defines the averaging over all presentations. Finally, the coefficients defined by Eq (7) were averaged over the subjects of groups 1 and 2 as

$$|{\langle {\bar{F}}_{\alpha ,\beta ,\delta }\rangle }_{\Delta t_{\text{I},\text{II},\text{III}}}|{}_{Gr.1,2}=\frac{1}{N_{Gr.1,2}}\sum_{N_{Gr.1,2}}^{}{\langle {\bar{F}}_{\alpha ,\beta ,\delta }\rangle }_{\Delta t_{\text{I},\text{II},\text{III}}},$$ (8)

where *N*_*Gr*.1_ and *N*_*Gr*.2_ are the number of participants in groups 1 and 2, respectively.

### Brain-computer interface for estimation and improvement of alertness

For estimation of human alertness in real time we built a brain-computer interface based on the brain response on the consecutively presented visual stimuli. Similar to the experiment described previously we used the EEG recorder Encephalan-EEGR-19/26 (Medicom MTD, Russia) supplemented by a special home-made developed acquisition software. A special library from Medicom MTD allowed us to access the data in real time with a sample rate of 250 Hz. The set of *N* = 5 EEG channels (P_3_, O_1_, P_*z*_, P_4_, O_2_) arranged according to “10-20” scheme. The wavelet spectrum of the EEG signals was calculated using a floating window of 2-sec length in the range between 4 Hz and 30 Hz. Each event was analyzed separately in alpha and beta frequency bands on a 1-sec interval preceding the presentation and followed by the moment of the stimulus appearance. A special digital trigger sent by the software together with the presentation of the stimuli initiated the calculation. As a result, the set of values *A*_*I*_, *A*_*II*_, *B*_*I*_, *B*_*II*_ were calculated for each presentation as

$$\begin{array}{c} A_{I,II}=\sum_{n=1}^{N}\int_{t\in \Delta t_{I,II}}\xi^{n}\left(t^{\prime }\right)dt^{\prime },\;\;\text{where}\;\;\;\xi^{n}\left(t\right)=\{\begin{array}{ccc} 1, & \text{if} & f_{max}^{n}\in \Delta f_{\alpha },\\ 0, & \text{if} & f_{max}^{n}\notin \Delta f_{\alpha }. \end{array} \end{array}$$ (9)

$$\begin{array}{c} B_{I,II}=\sum_{n=1}^{N}\int_{t\in \Delta t_{I,II}}\xi^{n}\left(t^{\prime }\right)dt^{\prime },\;\;\text{where}\;\;\;\xi^{n}\left(t\right)=\{\begin{array}{ccc} 1, & \text{if} & f_{max}^{n}\in \Delta f_{\beta },\\ 0, & \text{if} & f_{max}^{n}\notin \Delta f_{\beta }. \end{array} \end{array}$$ (10)

where *N* = 5 is the number of EEG channels and $f_{max}^{n}$ is the location of the maximal spectral component.

The obtained values were averaged over six presentations and the control characteristic *G*(*t*) was calculated as

$$\begin{array}{c} G\left(t\right)=\frac{\left(<A_{I}>-<A_{II}>\right)+\left(<B_{II}>-<B_{I}>\right)}{2}, \end{array}$$ (11)

where < … > means the average over six presentations.

The value of *G*(*t*) was calculated using Eqs (9)–(11) in real time. In the experiments with BCI, the feedback control was carried out in the form of a short sonic tone every time *G*(*t*) reached a threshold value, which was estimated for each subject individually, based on the previous value of *G*(*t*) averaged over a 4-min interval. The comparison between the current value of *G*(*t*) and its threshold value was made for every cube presentation.

## Results and discussion

The perception of an ambiguous image is associated with an increase in the electrical activity of neurons in the occipital lobe [73, 74]. Therefore, in the present work we analyze the EEG recordings from five channels (P_3_, O_1_, P_*z*_, P_4_, and O_2_) taken from the occipital lobe according to the scheme shown in Fig 2A. Below, in the same figure we present typical sets of the EEG trials recorded from these channels during visual perception.

In order to study the perception process, the EEG signals corresponding to each image presentation were partitioned into three segments: I, II, and III, as shown in Fig 2A. The segment I represented the EEG during the time interval preceding the cube presentation. The segment II corresponded to the time interval during the cube presentation until the observer pressed a button on the joystick. Finally, the segment III started immediately after the subject pressed the button and lasted for about 1 second. All EEG recordings were processed using the continuous wavelet transformation with the Morle wavelet function (for details see section “EEG analysis”). The wavelet power spectra were calculated for each segment in the frequency band Δ*f* ∈ [1, 40] Hz.

We found that the perception of ambiguous images can follow two different scenarios depending on the relationship between *α*-, *β*-, and *δ*-rhythms. In order to reveal the criteria for the selection of one or another scenario, we analyzed the EEG data separately for each subject and found that all perception trials can be classified into two groups of events with distinct spectral relationships, referred to as type-1 and type-2 events belonging respectively to the first and second scenarios.

In the first stage of the experiment, the location of the spectral component corresponding to the maximal value of the wavelet energy was estimated for every segment (I, II, III) and averaged over the whole session. Similar to the method of the event-related potential [32], the 3-sec traces of EEG (the structure of the trace is shown in Fig 2A) were extracted from the whole recording. For each trace the coefficient describing the location of the maximal spectral component was calculated by Eq (3). The obtained dependencies calculated for each segment were then lined up in time and averaged to diminish any brain activity unrelated to the stimulus. As a result, for each subject the dependence *L*(*f*, *t*) reflecting the dynamics of the main spectral component induced by the stimulus perception was obtained. In order to quantitatively characterize the obtained 2-D dependencies *L*(*f*, *t*) the coefficients $L_{\alpha }^{I}/L_{\alpha }^{II}$ and $L_{\beta }^{I}/L_{\beta }^{II}$ describing the variation of the spectral properties in alpha and beta bands during visual perception were calculated by Eq (4).

In Fig 2 we plot the coefficients $L_{\alpha }^{I}/L_{\alpha }^{II}$ and $L_{\beta }^{I}/L_{\beta }^{II}$ obtained for the group of 10 subjects, by circles and triangles, respectively. Having analyzed the obtained values we found that the subjects can be divided into two groups, according to two different scenarios of the perception process. Each subject was classified into one or another group based on a set of threshold values (dashed lines in Fig 2) defined by a > 40% decrease in alpha activity (line 1) and a > 20% increase in beta activity (line 2). The solid red boxes in Fig 2 highlight the subjects 2, 3, and 9 for which $L_{\alpha }^{I}/L_{\alpha }^{II}$ and $L_{\beta }^{I}/L_{\beta }^{II}$ satisfy the threshold values. These subjects were associated with the first scenario, while other subjects belonged to the second scenario.

Fig 2C and 2D show the typical distributions *L*(*f*, *t*) for the first (subject #9) and the second (subject #7) perception scenarios, respectively. The first scenario illustrated in Fig 2C) is characterized by a significantly low power of the 8-12 Hz oscillations (*α*-wave) during the cube observation (segment II) and a relatively high power of the 20-30 Hz oscillations (*β*-wave). The second scenario (Fig 2D) implies a strong contribution of the *α*-rhythm and much lower pronounced generation of the *β*-rhythm during all segments, while the low-frequency *δ*-rhythm (1-4 Hz) has low activity in segment II during the cube presentation.

**Fig 3:**
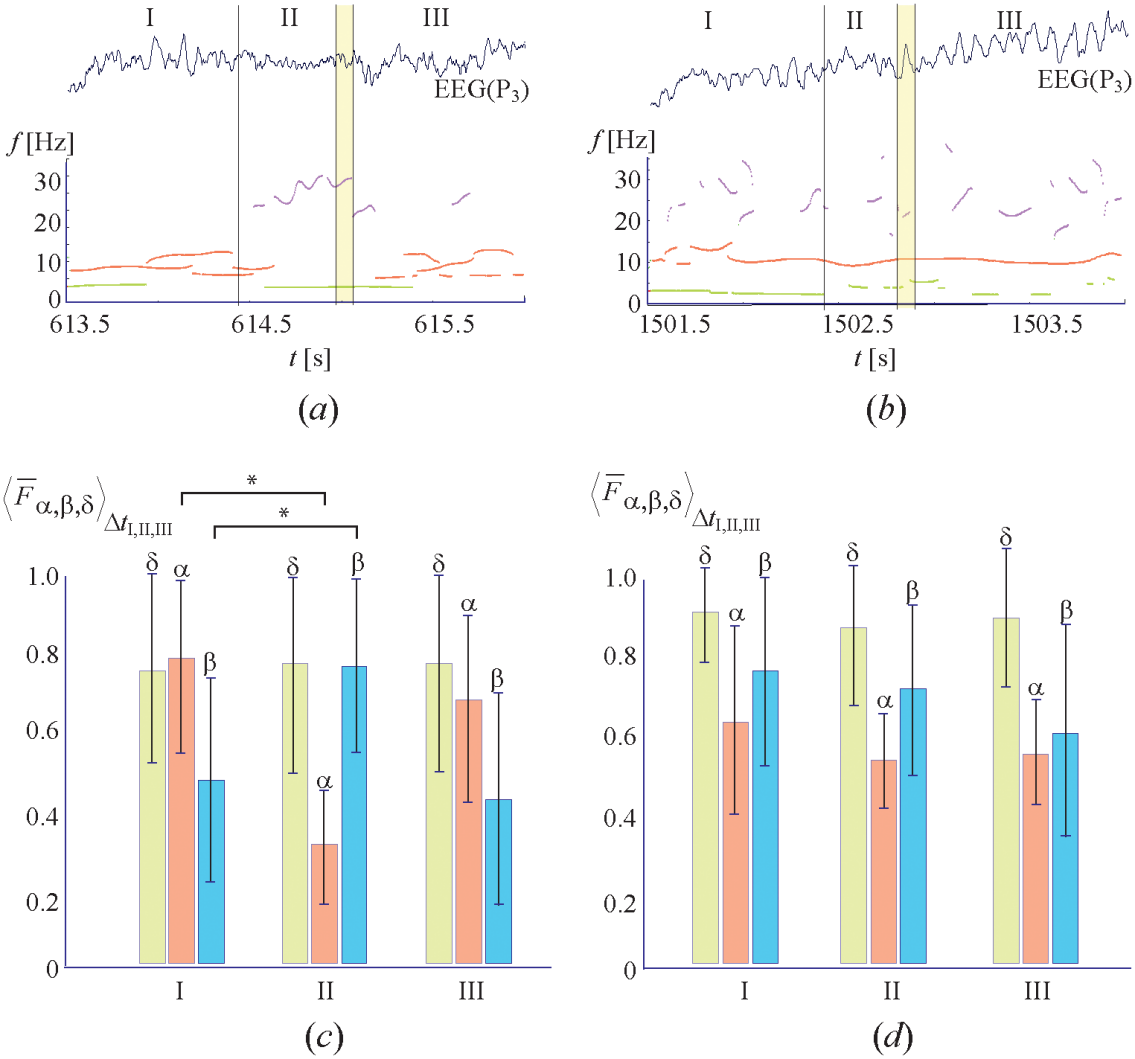
Spectral properties of two different perception scenarios. Upper row: Typical EEG trials associated with perception of ambiguous images illustrating (a) first and (b) second perception scenarios. The colored solid lines show the temporal dependences of the dominant frequencies of the first and second maximal spectral components during the perception. The line color indicates the frequency band within which these spectral components occur at the current moment of time: green refers to delta band (1-4 Hz), red to alpha band (8-12 Hz), and purple to beta band (20-30 Hz). The double vertical lines limit the time interval of the button pressing. Lower row: Coefficients 〈*F*_*α*,*β*,*δ*_〉 characterizing the location of the maximal spectral components averaged over all channels and time intervals Δ*τ*_I,II,III_ corresponding to different segments during perception for subjects belonging to (c) group 1 and (d) group 2. The error bars indicate standard deviations for each group. The horizontal bars with stars show significant differences in contributions of the alpha and beta components according to the statistical analysis using paired t-test.

The characteristic spectral features of type-1 and type-2 events are illustrated respectively in Fig 3A and 3B with EEG trials (upper curves) and dominant frequencies of *α*, *β*, and *δ* spectral components (colored solid lines) corresponding to the first and second maxima of the wavelet energy. The red, green, and purple colors indicate the frequency bands Δ*f*_*α*,*δ*,*β*_ belonging to *α*-, *β*-, and *δ*-rhythms, respectively. One can see from Fig 3A that type-1 events corresponding to the first scenario are characterized by the presence of a large-amplitude *α*-rhythm component in the wavelet spectrum during segments I and III, whereas in segment II this component is much lower and the *β* component is much higher. Unlike type-1 events, type-2 events (Fig 3B) corresponding to the second scenario are characterized by highly-pronounced *α*-rhythm during all perception stages. The two types of events were detected in all subjects, however the relation between the number of type-1 and type-2 events was different for each subject. This resulted in different averaged dependencies for each subject. For two subjects illustrated in Fig 2C and 2D the ratio between the number of type-1 and type-2 events was about 7:3 and 4:5, respectively.

All perception events were quantitatively classified into type-1 and type-2 events using the relation between spectral coefficients ${\langle F_{\alpha ,\beta ,\delta }\rangle }_{\Delta t_{\text{I},\text{II},\text{III}}}$ which described the contribution of *M* = 5 highest spectral components averaged over all EEG channels in each of three segments Δ*τ*_*I*,*II*,*III*_ corresponding to different perception stages (see Eq (6)). In particular, for all subjects the values of ${\langle F_{\alpha ,\beta ,\delta }\rangle }_{\Delta t_{\text{I},\text{II},\text{III}}}$ were averaged over 400 presentations and over participants belonging to group 1 and group 2 (see Eq (7)). The obtained results are shown in Fig 3C and 3D for group 1 and group 2, respectively. The error bars define the standard deviation of the considered values within all subjects in the group. One can see from Fig 3C that the subjects of group 1 exhibit a decrease in alpha activity from 0.81 ± 0.23SD in segment I to 0.36 ± 0.16SD in segment II and an increase in beta activity from 0.44 ± 0.22SD in segment I to 0.78 ± 0.21SD in segment II. According to the statistical analysis based on paired t-test such changes are judged as significant (*p*_*α*,*β*_ < 0.05) and marked by stars in Fig 3C. For the subjects in group 2 (Fig 3D), no significant changes were found in alpha and beta activity (*p*_*α*_ = 0.23, *p*_*β*_ = 0.36). Instead, the statistical analysis of ${\langle F_{\alpha }\rangle }_{\Delta t_{\text{I},\text{II}}}$ and ${\langle F_{\beta }\rangle }_{\Delta t_{\text{I},\text{II}}}$ for each subject, based on a large number of image perceptions, showed the existence of significant changes between the subjects in group I. So, each subject in group I demonstrated *p*_*α*,*β*_-value less than 0.05, whereas for the subjects in group II the *p*_*α*_ value varied from 0.085 to 0.43 and *p*_*β*_ from 0.175 to 0.492.

In order to find the reason for the occurrence of type-1 or type-2 event, we carried out the following experiment. All subjects were divided into two groups (10 subjects in each group) according to the degree of their motivation. The members of the first group (GROUP I) were financially motivated and instructed to focus their attention on every cube as much as possible until the experiment ended. In addition, for the participants from this group the experiments were arranged at the most convenient time for each subject. Instead, the members of the second group (GROUP II) were unpaid volunteering students and staff, subjected to experimental sessions at random times. They got the task to press a button based only on their first impression. It was supposed that due to the long duration of the session and high similarity of the cubes, the unmotivated subjects would lose their attention since it was not a special requirement.

**Fig 4:**
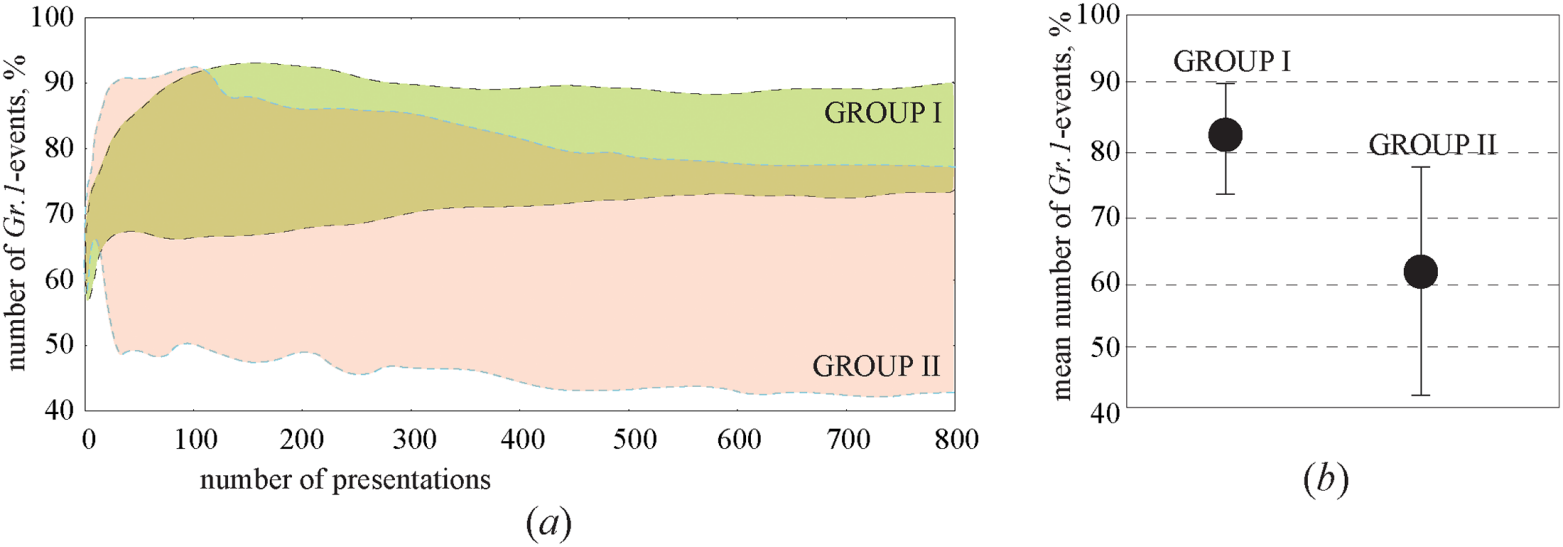
Effect of motivation. (a) Colored areas containing dependencies of the percentage of type-1 events on the number of the cube presentations for participants belonging to GROUP I (motivated subjects) and GROUP II (unpaid volunteers). (b) Percentage of type-1 events averaged over participants belonging to GROUP I (left circle) and GROUP II (right circle). The error bars show the standard deviation for each group.

Similarly to the first stage of the experiment, all participants were subjected to 40-min sessions during which the Necker cube was presented about 800 times. The number of type-1 and type-2 events was calculated according to the spectral properties described above. The results are shown in Fig 4A where the colors mark the areas containing dependencies of the percentage of type-1 events on the number of cube presentations for subjects belonging to GROUP I (upper region) and GROUP II (lower region). The first 500 presentations can be considered as transients characterized by considerable fluctuations. After the transient process was accomplished, the percentage of the events in each group remained constant. The solid circles in Fig 4B show the percentage of type-1 events averaged over the participants belonging to GROUP I (left circle) and GROUP II (right circle), and the error bars indicate the deviation of this value within each group. One can see that the percentage of type-1 events varied from 73% to 87% in GROUP I and from 47% to 77% in GROUP II, and the averaged percentages were ∼81% and ∼62%, respectively. Thus, our results demonstrated the experimental evidence that the participants belonging to GROUP I, affected by the financial incentive and having the opportunity to choose the most convenient time for the experiment, processed the visual object much more carefully than the subjects from GROUP II.

In order to study how ambiguity affects perception, we carried out an additional experiment with 10 extra volunteers. In this experiment each subject participated in two 20-min sessions. The design of these sessions was practically the same as the previous one, but the cube parameters were different. Namely, the cubes with low and high ambiguity were presented. The ambiguity was controlled by the contrast of the inner ribs. It was expected that higher ambiguity would increase the alertness so that the subject would make a decision more carefully.

**Fig 5:**
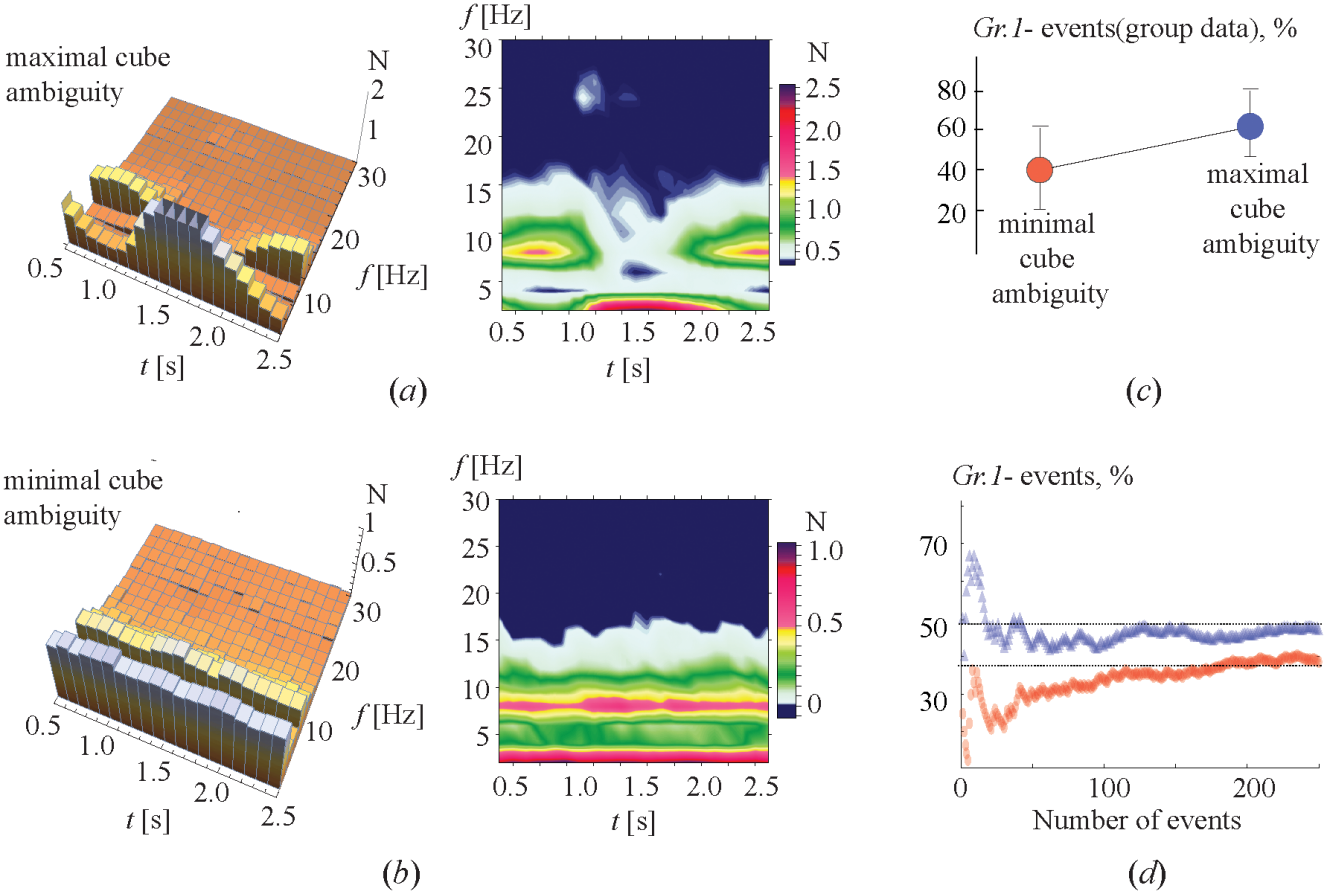
Effect of cube ambiguity. (a,b) 3-D histograms illustrating dominant frequencies of the maximal spectral component during sessions with (a) high and (b) low ambiguity for the same subject. (c) Percentage of type-1 events observed in sessions with low (left red circle) and high (right blue circle) ambiguity, averaged over all participants. The error bars indicate the deviation of these values among all participants. (d) Percentage of type-1 events as a function of the number of presentations of cubes with low (lower curve) and high (upper curve) ambiguity.

The results of the analysis are presented in Fig 5. The 3-D histograms in Fig 5A and 5B illustrate each of the two sessions for the same subject. As seen from these diagrams, the cubes with high ambiguity (Fig 5A) resulted in a much larger number of type-1 events than the cubes with low ambiguity. Fig 5C shows the percentage of type-1 events generated by the cubes with low and high ambiguity, averaged over all participants. The error bars indicate the deviation of these values among all participants. The dependencies of the percentage of type-1 events on the number of presentations are shown in Fig 5D. After some transients these dependences approach 30% and 50% for low and high ambiguity, respectively. The obtained results confirm our hypothesis, that an increase in cube ambiguity improves attention leading to an increasing number of type-1 events.

**Fig 6:**
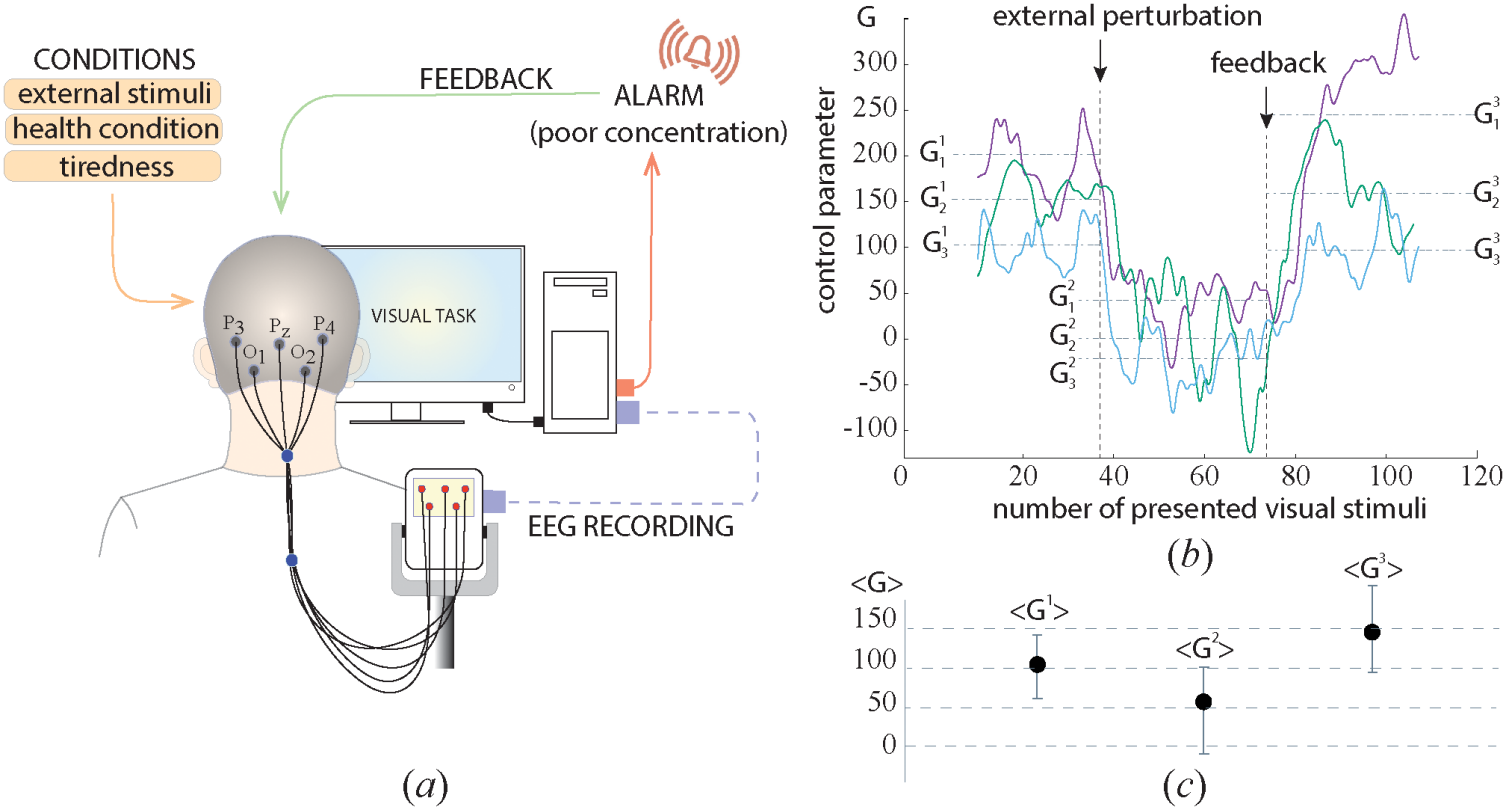
Brain-computer interface for estimation and control of alertness. (a) Schematical illustration of the experimental setup. (b) Control characteristics *G*_1,2,3_(*t*) describing the degree of alertness during the processing of visual stimuli, obtained from three subjects of the group. The vertical dashed lines indicate the moments of time when the external disturbance (*t*_*EP*_) was applied and the feedback message about the attention decrease (*t*_*FB*_) was sent. The horizontal dash-dotted lines indicate the values of $G_{1,2,3}^{1}$, $G_{1,2,3}^{2}$, $G_{1,2,3}^{3}$ calculated by averaging *G*_1,2,3_(*t*) over time intervals *t* < *t*_*EP*_, *t*_*EP*_ > *t* > *t*_*FB*_ and *t* > *t*_*FB*_. (c) Values of $G_{1,2,3}^{1}$, $G_{1,2,3}^{2}$ and $G_{1,2,3}^{3}$ averaged over the group of eight subjects. The error bars indicate the standard deviation of these values among all participants.

Having summarized the results of the experiments described above, one can see that the degree of human alertness can vary during perception of bistable images depending on the motivation level and the task complexity. The degree of alertness in turn can be estimated by the ratio between the number of perception trials classified into type-1 and type-2 events, according to the EEG spectral properties. Since every particular event can be immediately classified into one of the two types, it is possible to estimate the changes in the degree of alertness in real time. In order to check this possibility, we developed the brain-computer interface (BCI) for estimation and control of human alertness. The experimental setup is shown in Fig 6A. The interface was based on the EEG recorder Encephalan-EEGR-19/26 (Medicom MTD, Russia) supplemented by a special home-made developed acquisition software. The degree of alertness was measured in real time by calculating *G*(*t*) using Eq (11) based on the spectral analysis of the EEG signal (see Materials and methods for details).

The BCI was experimentally tested on three volunteers. In this experiment, each subject participated in three 4-min subsequent sessions. The experimental results are illustrated in Fig 6B and 6C. The left and right arrows indicate, respectively, the moments of time, *t*_*EP*_ and *t*_*FB*_, when the external influence and the feedback control were switched on. These moments divided the experiment into three sections. During the first section (*t* < *t*_*EP*_), the subject performed the task in the absence of external influence. One can see that *G*(*t*) fluctuated near a certain mean value of $G_{1,2,3}^{1}$, individual for each subject. The second section (*t*_*EP*_ < *t* < *t*_*FB*_) included the external influence on the subject in the form of an additional cognitive task. It is easy to see that when the external influence took place, the value of *G*(*t*) sharply decreased for all subjects and oscillated near the mean value $G_{1,2,3}^{2}$, significantly lower than the mean in the first section. Finally, the third section started at (*t* = *t*_*FB*_) demonstrated the effect of the feedback control, when the subject received a short audio stimulus, sent each time the value of *G*(*t*) fell below the threshold level which was estimated for each subject, based on the values of $G_{1,2,3}^{1}$. One can see that in the presence of the feedback contro, *G*(*t*) significantly increased for all subjects and oscillated near the mean values $G_{1,2,3}^{3}$. It is important to note that a significant change in *G*(*t*) was observed within a relatively short time interval (less than 30 seconds) during which the visual stimulus was presented about 5 times. This means that the significant loss of attention can be promptly detected and controlled in real time.

## Conclusion

The results of this study have confirmed our hypothesis that the brain electric activity while processing an optical illusion can be classified into two distinct scenarios depending on the degree of attention of the observer, which in turn is affected by the motivation of and task complexity. We have come to this conclusion based on the consideration of particular features of EEG signals in alpha (8–12 Hz) and beta (20–30 Hz) bands before and during the presentation of ambiguous images. Our finding of the criteria for the classification of each percept into one or another scenario allowed us to measure the ratio between the occurrence of the first and the second scenarios in real time, by analyzing spectral properties of multichannel EEG data. We have shown that the degree of attention can be estimated by measuring the ratio between the percentages for the occurence of one or another scenario. The effects of motivation and task complexity have also been analyzed.

Our hypothesis has been verified in series of electrophysiological experiments. First, bistable images were randomly presented to different subjects in similar conditions. Then, we performed statistical analysis of the brain activity of every subject in alpha and beta bands during the transition from a “ready” state (before image exposition) to the perception (image exposition). All participates have been divided into two groups according to the effective destruction of alpha waves and the increase in beta activity. Based on the obtained results we have revealed distinguished features for the first and second scenarios and calculated the ratio between individual perceptions, which obeyed these scenarios for every participant. After that, we have performed similar experiments with two groups of subjects, financially motivated participants and unpaid volunteers. Using the obtained EEG data we have calculated the number of events belonging to the first and second scenarios and compared these values for two groups. Next, we have carried out experiments with each group of participants by presenting images with high and low ambiguity, and analyzing the relation between the first and second scenarios. Finally, we have developed a brain-computer interface and demonstrated how the degree of attention can be estimated in real time and how it can be controlled by a feedback signal using external stimulation.

The EEG trials associated with perception of ambiguos images have been classified as type-1 and type-2 events depending on the relation between alpha and beta waves. The type-1 events exhibited a transition of the spectral energy from alpha to beta frequency band with a simultaneous increase in the interaction between remote regions of the occipital lobe in generation of beta-wave activity. The type-2 events were characterized by strong contribution of alpha wave before, during, and after image presentation, as well as the participation of remote regions of the occipital lobe in the generation of this rhythm. Although these types of events have been detected in all subjects, the relation between them was different, which determined the scenario for visual perception. The results of our experiments have shown that the choice between one or another scenario depended on the degree of motivation and alertness of the observer; strong motivation and high alertness caused mainly the first scenario.

The revealed phenomena can be associated not only with visual perception, but also with other types of cognitive tasks which require a high level of alertness. The observed scenario can be detected automatically using a real-time processing of the EEG signals, which can have important applications in monitoring and controlling human attention and alertness during tasks which require substantial attention, e.g., air traffic control, monitoring nuclear power plants, development of training programs and tests of human psychological conditions. Unlike the majority of publications on the topic of motivation and alertness, our approach deals with each individual perception and can classify it into different scenarios according to spectral properties of the recorded EEG. This opens the possibility to estimate the variation of the degree of human attention in time, which is necessary for the development of systems for control and training.

In order to prove our approach we have built the prototype of a noninvasive brain-computer interface for estimation and control of human alertness in real time. The device was based on the EEG recorder supplemented by a special home-made developed acquisition software. When the subject lost attention, the sound signal was given for sharpening of attention. The devices based on the developed BCI can find applications for pilots, military, long-distance drivers, and people of other professions requiring increased attention.
